# Correction to: Estimating the redox state of the plastoquinone pool in algae and cyanobacteria *via* OJIP fluorescence: perspectives and limitations

**DOI:** 10.1007/s11120-026-01203-7

**Published:** 2026-02-16

**Authors:** Tomáš Zavřel, Anne-Christin Pohland, Tobias Pfennig, Anna Barbara Matuszyńska, Szilvia Z. Tóth, Gábor Bernát, Jan Červený

**Affiliations:** 1 Department of Adaptive Biotechnologies, Global Change Research Institute, Czech Academy of Sciences, Brno, Czechia; 2 Bio-Inspired Energy Conversion, Technical University Darmstadt, Darmstadt, Germany; 3 Computational Life Science, Department of Biology, RWTH Aachen University, Aachen, Germany; 4 Cluster of Excellence on Plant Sciences, Heinrich Heine University Düsseldorf, Düsseldorf, Germany; 5 Laboratory for Molecular Photobioenergetics, Institute of Plant Biology, HUN-REN Biological Research Centre, Szeged, Hungary; 6 Aquatic Botany and Microbial Ecology Research Group, HUN-REN Balaton Limnological Research Institute, Tihany, Hungary

**Correction: Photosynthesis Research (2026) 164:6**

10.1007/s11120-025-01194-x

The original article has been corrected. An uncorrected proof was originally published and has been replaced with the final version of the article.

